# Potential beneficial effects of oral administration of isoflavones in patients with chronic mountain sickness

**DOI:** 10.3892/etm.2013.1388

**Published:** 2013-11-07

**Authors:** JIANHUA CUI, LIANG GAO, HAIJUN YANG, FULING WANG, CHUNHUA JIANG, YUQI GAO

**Affiliations:** 1 Research Center of PLA for Prevention and Treatment of High Mountain Sickness, The 18th Hospital of PLA, Yecheng, Xinjiang 844900, P.R. China; 2 Department of Pathophysiology and High Altitude Physiology, College of High Altitude Military Medicine, Third Military Medical University, Chongqing 400038, P.R. China; 3 Key Laboratory of High Altitude Medicine, Ministry of Education, Chongqing 400038, P.R. China; 4 Key Laboratory of High Altitude Medicine, PLA, Chongqing 400038, P.R. China

**Keywords:** high altitude, chronic mountain sickness, isoflavone, erythrocytosis, pulmonary hemodynamics

## Abstract

Soy isoflavones (Ifs), which are natural phytoestrogens, have beneficial effects in cardiovascular disease. We have previously shown that genistein, the most active component of Ifs, inhibits pulmonary vascular structural remodeling and right ventricular hypertrophy induced by chronic hypoxia in male Wistar rats. This study aimed to evaluate the effects of Ifs on right ventricular and pulmonary hemodynamics in individuals with chronic mountain sickness (CMS). Twenty-eight male patients living on the Qinghai-Tibetan plateau (5,200 m) who were suffering from CMS were treated orally with Ifs (20 mg, twice daily) for 45 days. Physiological and plasma biochemical indices, hematology and echocardiography were investigated. It was observed that 45 days of treatment with Ifs significantly increased blood oxygen saturation and markedly decreased the CMS score and heart rate (all P<0.05) of the subjects. Following treatment with Ifs, hematocrit (P<0.05), hemoglobin concentration (P<0.01) and plasma levels of malondialdehyde (P<0.05) were significantly decreased, while plasma levels of nitric oxide (P<0.01) and the plasma activity of nitric oxide synthase (P<0.01) and superoxide dismutase (P<0.01) were markedly increased compared with the respective values obtained prior to treatment with Ifs. The echocardiography results showed that Ifs significantly decreased the main pulmonary artery diameter (P<0.05), right ventricular end-diastolic anteroposterior diameter (P<0.01), right ventricular end-diastolic trans diameter (P<0.01), right ventricular anterior wall (P<0.01) and right ventricular outflow tract (P<0.01). These results indicate the potential beneficial effects of Ifs in the reduction of excessive erythrocytosis, the alleviation of oxidative damage and the amelioration of right ventricular index and pulmonary hemodynamics in CMS.

## Introduction

Chronic mountain sickness (CMS) or Monge's disease, first described by Carlos Monge Medrano in Peru in 1925 (1), is characterized by excessive erythrocytosis (a hemoglobin level of ≥19 g/dl in females or ≥21 g/dl in males) in combination with chronic hypoxemia; in certain cases pulmonary hypertension is also apparent (2–4). Severe pulmonary hypertension may evolve to cor pulmonale, resulting in congestive heart failure. Therefore, CMS frequently leads to right cardiac failure and/or neurological disorders (3–5). The clinical signs in patients with CMS include breathlessness and/or palpitations, sleep disturbance, cyanosis, dilatation of veins, paresthesia, headache and tinnitus (2,4). On the Qinghai-Tibetan plateau, the incidence of CMS is 2.43–37.5% (3,000–4,700 m) and increases with elevation (4,6). Furthermore, the prevalence of CMS is significantly increased in migrants compared with that in the native population of high altitudes, and the disease occurs more frequently in males than in females (7–9). CMS is a severe public health problem for the Qinghai-Tibetan plateau, which has >60 million people in residence (4).

Despite a number of trials with medroxyprogesterone, enalapril and almitrine, no pharmacological treatment is currently widely used to impede the progressive loss of adaptation to chronic hypoxia (10–13). Acetazolamide (Acz) is useful for reducing hematocrit and improving pulmonary circulation in CMS. However, Acz has adverse effects, including paresthesia and diuresis (14). Therefore, the only management for CMS, at present, is repetitive bloodletting or the displacement of dwellers to lower altitudes. The latter option has an important negative social and economic impact on the development of high-altitude regions.

Excessive erythropoiesis in CMS is a process that is hormonally regulated (15,16). High serum testosterone levels are correlated with excessive erythrocytosis in males at high altitude, and female sex hormones suppress polycythemic and cardiopulmonary responses *in vivo* during chronic hypoxic exposure (16,17). It is generally accepted that an excessive number of red blood cells in the vessels is likely to increase blood viscosity, thereby inducing pulmonary hypertension and even cardiac failure. However, the underlying mechanism of pulmonary hypertension in CMS is only partially understood. Data from Bailey *et al*(18) indicated that increased pulmonary arterial systolic pressure at high altitude was correlated with a reduction in pulmonary nitric oxide (NO) bioavailability (18). Therefore, the inhibition of erythropoiesis and the increase of circulating NO levels are likely to alleviate the clinical signs of patients with CMS.

Soy isoflavones (Ifs) are the most studied and most potent phytoestrogens, and are predominantly present in soy-based foods (19). Genistein and daidzein, the two most abundant Ifs (19), have chemical structures similar to that of estradiol (E2) and exert estrogen-like effects (20), which are able to inhibit excessive erythropoiesis. Experimental studies have shown the beneficial effects of Ifs on the production of NO in endothelial cells, which include inhibition of the proliferation of vascular smooth muscle and reduction of the tissue damage caused by reactive oxygen species (ROS) (21,22). Animal studies have also indicated that genistein and daidzein, through estrogen receptor (ER)-independent mechanisms, restore endothelial cell function by increasing NO production and the protection of NO from O_2_^•−^driven inactivation (23). In addition, our previous studies showed that treatment with genistein inhibited pulmonary vascular structural remodeling and right ventricular hypertrophy induced by chronic hypoxia (5,000 m, 21 days) in male Wistar rats without adverse effects, such as feminizing and oncogenic effects (24–26).

Therefore, in the present study it was hypothesized that Ifs may reduce excessive erythropoiesis and ameliorate right ventricular index and pulmonary hemodynamics in CMS. The study aimed to observe the effects of Ifs in patients with CMS and to evaluate the treatment of CMS.

## Materials and methods

### Subjects and procedure

Twenty-eight male patients suffering from CMS, who were living on the Qinghai-Tibetan plateau (5,200 m), gave their informed written consent for this study, which was approved by the Ethics Committee of the Third Military Medical University (Chongqing, China). The Ethics Committee of the Third Military Medical University refused to permit a study design where no treatment was administered to patients who had been diagnosed with CMS with malaise. Therefore, placebo treatment was not given to the patients with CMS. This study was only designed to observe the effects of Ifs on patients with CMS by comparing various parameters prior to and following treatment. All subjects, who were born and previously lived in a low-altitude area, were migrants of the Qinghai-Tibetan plateau (5,200 m), had been residing at the plateau >10 months and did not travel to lower altitudes (<5,000 m). All patients had a hemoglobin concentration ≥21 g/dl and were working at the same labor intensity. A complete clinical evaluation, including an evaluation of ventilatory function and cardiac ultrasonography, was performed prior to inclusion in the study to exclude subjects with cardiac or pulmonary diseases. The age, weight and height of the subjects were 20.86±1.53 years, 66.36±6.59 kg and 1.76±0.35 m, respectively. The CMS clinical score was calculated according to a consensus statement on chronic high-altitude diseases (2) and was based on the following symptoms: breathlessness and/or palpitation, sleep disturbance, cyanosis, dilatation of veins, paresthesia, headache and tinnitus. Finger blood oxygen saturation (SaO_2_) and heart rate (HR) were measured using a TuffSat handheld oximeter (GE Healthcare, Fairfield, CT, USA) following a 15-min rest in the sitting position. Subjects were treated orally with Ifs capsules (containing 20 mg mixed Ifs, twice daily; Department of Traditional Chinese Medicine Preparation, Xinqiao Hospital, Third Military Medical University, Chongqing, China) for 45 days. All measurements were obtained at low altitude on two occasions, prior to and following treatment with Ifs.

### Hematological status

Anticoagulated peripheral venous blood samples were obtained from subjects at 5,200 m in the sitting position, immediately subsequent to the subjects waking up in the morning. Hemoglobin concentration was obtained using cyanide measurements and a duplication was performed for each sample. The measurements for hematocrit were obtained using Symex pocH-80i automatic blood cell analyzer (Sysmex, Kobe, Japan). Following centrifugation at 1,000 × g for 10 min, the plasma was collected and stored at −20ºC. The levels of plasma NO, nitric oxide synthase (NOS), superoxide dismutase (SOD) and malondialdehyde (MDA) were measured using corresponding chemical colorimetry reagent kits (Nanjing Jiancheng Bioengineering Institute, Nanjing, China), in accordance with the manufacturer's instructions.

### Echocardiographic measurements

Echocardiography was performed with a 2.5-MHz transducer (Logiq Book XP, GE Healthcare) to evaluate the main pulmonary artery diameter (MPA), right ventricular end-diastolic anteroposterior diameter (RVEAD), right ventricular end-diastolic trans diameter (RVETD), right ventricular anterior wall (RVAW) and right ventricular outflow tract (RVOT) by a single experienced echocardiographer, as previously described (27).

### Statistical analysis

Data are expressed as the mean ± standard deviation. The analyses were performed using a Student's t-test. P<0.05 was considered to indicate a statistically significant difference.

## Results

### Effects of treatment with Ifs on physiological outcomes

Following 45 days of treatment with Ifs, SaO_2_ was significantly increased by 2.04% (P<0.05), the CMS score was decreased by 1.07 points (P<0.01) and HR was decreased by 4.17 beats/min (P<0.05) compared with the values prior to treatment (Table I). These changes clearly indicated an improvement in hypoxemia and a reduction of clinical malaise in patients with CMS.

### Effects of treatment with Ifs on hematological outcomes

The number of polycythemic individuals (hemoglobin ≥21 g/dl) decreased from 28 to 17 following treatment with Ifs. In addition, treatment with Ifs significantly decreased the hematocrit by 4% (P<0.05), hemoglobin concentration by 1.06 g/dl (P<0.01) and plasma levels of MDA by 1.16 nmol/ml (P<0.05), and markedly increased the plasma levels of NO by 7.70 μmol/l (P<0.01) and the activities of NOS and SOD by 7.19 and 10.63 U/ml, respectively (P<0.01 for each; Table II). These changes indicated that Ifs reduced the hemoglobin concentration and increased the plasma NO levels and antioxidant capacity in patients with CMS.

### Effects of treatment with Ifs on the cardiac and pulmonary circulation

Table III shows the echocardiographic measurements of the patients with CMS. Forty-five days of treatment with Ifs significantly decreased MPA by 1.38 mm (P<0.05), RVEAD by 1.73 mm, RVETD by 3.00 mm, RVAW by 0.50 mm and RVOT by 2.19 mm compared with the values prior to treatment (all P<0.01). These results suggested an amelioration in the pulmonary hemodynamics and right ventricular index in patients with CMS.

## Discussion

CMS is an excessive polycythemia that is common among high-altitude residents, particularly in migrants (2). According to the prevalence of CMS, millions of residents on the Qinghai-Tibetan plateau suffer from CMS. Therefore, these residents may benefit from a treatment that reduces their hemoglobin concentration and pulmonary arterial pressure.

The physiopathology of CMS has been attributed to the following sequence: hypoxemia, excessive erythropoiesis and pulmonary hypertension (4,7). In the present study, following treatment with Ifs, the hematocrit and hemoglobin concentrations of the subjects were significantly decreased compared with the values prior to treatment. Therefore, the number of polycythemic individuals (hemoglobin ≥21 g/dl) decreased from 28 to 17 following treatment with Ifs. Moreover, 45 days of treatment with Ifs significantly increased SaO_2_ and decreased HR and the CMS score compared with the values prior to treatment, which indicated a reduction in hypoxemia and clinical indisposition. In addition, Ifs significantly reduced MPA, RVEAD, RVETD, RVAW and RVOT. Therefore, these results indicated improvements in hypoxia and clinical indisposition, and an amelioration of the right ventricular index and pulmonary hemodynamics in patients with CMS following treatment with Ifs. Furthermore, these results were consistent with our previous observations in rats, which showed the preventive effects of phytoestrogens on chronic hypoxic pulmonary hypertension and right ventricular hypertrophy (25,26). In addition, there were no adverse effects, such as diuresis, paresthesia and heartburn, following treatment with Ifs in the present study. Data from Hamilton-Reeves *et al*(28) and our own laboratory showed that Ifs elicited no effects on reproductive hormones in men or male rats (26,28). Ifs may have a more extensive applications in patients with CMS compared with Acz, which exhibits beneficial and adverse effects.

Furthermore, the present study demonstrated that treatment with Ifs significantly decreased plasma MDA levels and increased SOD activity, indicating an attenuation of oxidative damage in patients with CMS. This may result in the relief of malaise in patients with CMS during long-term exposure to high-altitude hypoxia. Marked increases in the plasma activity of NOS and circulating NO levels were observed following 45 days of treatment with Ifs compared with the values prior to treatment. NO has been demonstrated to be important in adaptation or acclimatization to high altitude. Scherrer *et al*(29) revealed that NO reduced pulmonary arterial pressure and improved oxygen saturation among patients suffering from high-altitude pulmonary edema (29). Furthermore, total NO has been demonstrated to be negatively correlated with pulmonary arterial systolic pressure and positively correlated with pulmonary blood flow and oxygen delivery at 4,200 m (30). In age and biomarker-adjusted logistic regression, NOS was also shown to be inversely predictive of CMS (31). In the present study, the increased plasma activity of NOS and circulating NO levels, induced by 45 days of treatment with Ifs, relaxed small pulmonary arteries and reduced MPA and right ventricular ejection resistance. These observations were consistent with the results from our previous studies, in which treatment with genistein, the most active form of Ifs, inhibited pulmonary vascular structural remodeling and right ventricular hypertrophy induced by chronic hypoxia (5,000 m, 21 days) in male Wistar rats (25,26).

In conclusion, the present study demonstrated the potential beneficial effects of Ifs in reducing excessive erythrocytosis, alleviating oxidative damage and ameliorating right ventricular index and pulmonary hemodynamics in CMS. We propose that Ifs are a potential low-cost treatment for CMS. Studies with a control group and a dose-effect analysis, and large-scale trials are required to evaluate the feasibility of the implementation of treatment for CMS in the Tibetan region.

## Figures and Tables

**Table I:** Effects of treatment with isoflavones on the physiological parameters of patients with CMS (n=28).

Time-point	CMS score (points)	Heart rate (beats/min)	SaO_2_ (%)
Prior to treatment	4.68±1.47	92.46±10.97	81.07±3.39
Following treatment	3.61±2.18	88.29±10.46	83.11±3.97
P-value	0.007	0.042	0.028

Values represent the mean ± standard deviation. P-value, comparison between values prior to and following treatment with isoflavones. CMS, chronic mountain sickness; SaO_2_, blood arterialized oxygen saturation.

**Table II:** Effects of treatment with isoflavones on the hematological outcomes of patients with CMS (n=28).

Time-point	Hematocrit	Hemoglobin (g/dl)	MDA (nmol/ml)	SOD (U/ml)	NOS (U/ml)	NO (μmol/l)
Prior to treatment	0.71±0.08	22.24±0.78	4.81±2.09	68.10±18.56	40.43±13.61	47.50±14.29
Following treatment	0.67±0.05	21.18±1.05	3.65±2.11	78.73±14.64	47.62±10.87	55.20±14.10
P-value	0.012	<0.001	0.012	<0.001	0.002	0.004

Values represent the mean ± standard deviation. P-value, comparison between values prior to and following treatment with isoflavones. CMS, chronic mountain sickness; MDA, malondialdehyde; SOD, superoxide dismutase; NOS, nitric oxide synthase; NO, nitric oxide.

**Table III:** Effects of treatment with isoflavones on the cardiac and pulmonary circulation of patients with CMS (n=28).

Time-point	MPA (mm)	RVEAD (mm)	RVETD (mm)	RVAW (mm)	RVOT (mm)
Prior to treatment	23.38±2.70	23.39±3.12	30.71±3.75	5.89±1.22	33.89±3.59
Following treatment	22.00±2.31	21.66±2.49	27.71±3.38	5.39±0.79	31.70±3.37
P-value	0.011	<0.001	<0.001	0.010	0.002

Values represent the mean ± standard deviation. P-value, comparison between values prior to and following treatment with isoflavones. CMS, chronic mountain sickness; MPA, main pulmonary artery diameter; RVEAD, right ventricular end-diastolic anteroposterior diameter; RVETD, right ventricular end-diastolic trans diameter; RVAW, right ventricular anterior wall; RVOT, right ventricular outflow tract.

## Abbreviations

CMS: chronic mountain sickness
Acz: acetazolamide
Ifs: isoflavones
NO: nitric oxide
NOS: nitric oxide synthase
SOD: superoxide dismutase
MDA: malondialdehyde
MPA: main pulmonary artery diameter
RVEAD: right ventricular end-diastolic anteroposterior diameter
RVETD: right ventricular end-diastolic trans diameter
RVAW: right ventricular anterior wall
RVOT: right ventricular outflow tract
